# Non-specific protein modifications may be novel mechanism underlying bioactive phytochemicals

**DOI:** 10.3164/jcbn.17-113

**Published:** 2018-02-07

**Authors:** Akira Murakami

**Affiliations:** 1 Food Hormesis Laboratory, Department of Food Science & Nutrition, School of Human Science & Environment, Research Institute for Food and Nutritional Sciences, University of Hyogo, 1-1-12 Shinzaike-Honcho, Himeji, Hyogo 670-0092, Japan

**Keywords:** hormesis, zerumbone, target molecule, protein quality control, mechanism

## Abstract

In a variety of experimental models, dietary phytochemicals have been demonstrated to exhibit pronounced and versatile bioactivities. Importantly, the possibility of such phytochemicals for human application has been supported in part by epidemiological surveys, which have demonstrated that frequent ingestion of vegetables and fruits containing abundant phytochemicals lowers the risk of onset of various diseases. However, the action mechanisms underlying those dietary phytochemical activities remain to be fully elucidated. For example, even though the anti-oxidant effects of natural polyphenols have long received widespread attention from food scientists, their roles in and contribution to those bioactivities remain controversial because of their poor bioavailability, resulting in extremely low concentrations in the bloodstream. Meanwhile, another important question is why phytochemicals have beneficial effects for animals, including humans, since they are biosynthesized by plants as compounds necessary for adaptation to environmental stress. In regard to that fundamental question, we recently reported novel and unique mechanisms of action of zerumbone, a sesquiterpene with anti-inflammatory and chemopreventive properties. This agent was found to partially exhibit bioactivity through its non-specific interactions with cellular proteins. More strikingly, a non-specific protein binding action of zerumbone was revealed to partially contribute to its anti-inflammatory functions via activation of heat shock factor 1. The present review article highlights and introduces our recent findings regarding the proteo-stress-mediated mechanisms of this phytochemical, along with the concept of hormesis.

## Phytochemicals are Plant Secondary Metabolites

Environmental stress in plants can be classified into physical (e.g., intense sunlight), chemical (invasion by other plants and microorganisms), and biological (e.g., herbivorous animals) types. Reactions to various types of stress are thought to be generated to gain biological predominance over other species for survival in the process of natural selection. Thus, the adaptation capacity of an organism is a critical factor to pass genes to the next generation. In this context, plants have an essential disadvantage to fight against animal-derived stress, because they cannot move or run away from biological enemies or to avoid stress stimuli.

**Fig. 1:**
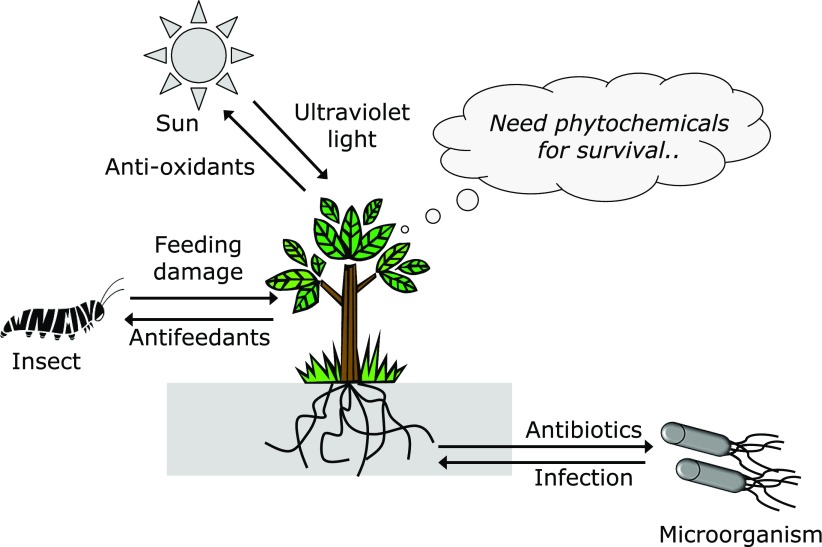
Roles of phytochemicals in stress adaptation. Plants are exposed to continuous environmental stressors that induce them to biosynthesize secondary metabolites, such as antioxidants, antifeedants, and antibiotics, as well as others, without which they would be destroyed by the stressors.

Interestingly, plant secondary products not essential for their survival have been shown to play important and primary roles in adaptation mechanisms (Fig. 1). For example, lignins, which comprise an integral part of secondary cell walls, are well known to serve as a physical barrier against invading organisms.^(1)^ Additionally, polyphenols, including flavonoids and simple phenolic acids, have been demonstrated to have anti-oxidant functions, which are useful and effective for protection against ultraviolet (UV) light-induced oxidative damage, because those phytochemicals possess biological chromophores that specifically absorb UV light. Moreover, terpenoids, especially volatile ones abundantly present in herbs and citrus, have unique roles as ‘infochemicals’. These are produced in response to feeding damage caused by insects and others, and can convey a danger sign to neighboring plants for species conservation.^(2)^ Using a different tactic, sulfur compounds selectively occurring in cruciferous plants, such as isothiocyanates (ITCs) and diallyl sulfide, have unpleasant odors and are chemical weapons with substantial toxicity to biological invaders.^(3)^

## Biological Functions and Recently Identified Mechanisms

Ample evidence showing that phytochemicals exhibit a wide array of physiological activities in experimental animal models as well as humans has been presented. For example, curcumin, a major component of turmeric (*Curcuma longa*), has long been reported to have multiple preventive effects on development of lifestyle-related diseases.^(4,5)^ In addition to its anti-oxidative properties, recent studies have revealed novel and unique mechanisms of action of this agent. miRNA, discovered in the late 20th century, is a unique class of small RNAs derived from the non-coding DNA regions of a gene, such as intron, and 5'- and 3'-untranslated region (UTRs), which play multiple roles in cell regulation, including proliferation, differentiation, and apoptosis. Expression of this class of RNA is tightly regulated by environmental conditions, including temperature,^(6)^ pressure,^(7)^ hormones,^(8)^ endotoxins^(9)^ and environmental toxins,^(10)^ to maintain homeostasis. Several recent studies have revealed that some phytochemicals modulate miRNA expression to exhibit their physiological functions. Lelli *et al.*^(11)^ presented a review article describing the anti-cancer activities of curcumin that occur via modification of miRNAs such as miR-21, which plays important roles in cell cycle regulation and apoptosis through down-regulation of phosphatase and tensin homolog deleted from chromosome 10 and the programmed cell death protein. Along a similar line, down-regulation of miR-21, miR-17-5p, miR-20a and miR-27a, and up-regulation of miR-34 have been shown to be involved in the anti-carcinogenesis effects of curcumin.^(12)^

In other recent studies, bioactive phytochemicals have been demonstrated to be epigenetic modifiers.^(13,14)^ Epigenetics is defined as heritable changes related to the activities of gene expression and suppression that occur without alteration of the DNA sequence. These include DNA methylation and histone modifications, such as acetylation, methylation, ubiquitylation, phosphorylation, sumoylation, ribosylation and citrullination.^(15–17)^ Meanwhile, human telomerase reverse transcriptase (*hTERT*), a catalytic subunit of telomerase, has been shown crucial for sustaining telomere chain length and tumor formation, the latter occurring through epigenetic regulation of the estrogen receptor.^(14)^ Green tea catechins such as (–)-epigallocatechin-3-gallate (EGCG) have been found to inhibit breast cancer cell growth, transcription of hypomethylation of the *hTERT* promoter region, and histone deacetylation via modulations of DNA methyltransferases (DNMT) and histone acetyltransferases (HAT).^(14)^ Similarly, quercetin, a flavonol present in onions and other vegetables, was reported to activate HAT and inhibit HDAC to exert epigenetic modulation for inducing Fas ligand-mediated apoptosis in human leukemia HL-60 cells.^(14)^ Moreover, curcumin was found to restore the expression of suppressor of cytokine signaling (SOCS) 1 and SOCS3 via inhibition of histone deacetylase activity, leading to increased histone acetylation in the SOCS1 and SOCS3 promoter regions in myeloproliferative neoplasm K562 cells.^(18)^

Molecular targeting of phytochemicals has been rigorously utilized in various studies since discovery of the receptor for EGCG, which exhibits multiple functions.^(19–24)^ Recently, many have uncovered molecular targets of phytochemicals that directly mediate their biological and physiological functions. For example, Singh *et al.*^(25)^ conducted extensive screening of a total of 803 phytochemicals to examine their binding affinity for androgen receptor (AR) and found that the prenylated flavonoid isobavachin exhibited the highest affinity (–13.73 kcal/mol), followed by gabranin, athocyanin, and eiosemation. Also, β-secretase 1 (BACE1) has been noted to play a crucial role in development of Alzheimer’s disease. Chakraborty *et al.*^(26)^ designed a multi-tier screening protocol to identify potential BACE1 inhibitor and found that hesperidin, a flavonoid in citrus fruits, docks close to catalytic aspartate residues, then orients itself for blocking the cavity opening and thereby disturbs substrate binding. Furthermore, an effective experimental approach that utilized click chemistry identified that the novel molecular target of xanthohumol, an *enone*-containing electrophilic chalcone from hops (*Humulus lupulus* L.), was glucose-6-phosphate dehydrogenase,^(27)^ a finding that may have some associations with the anti-obesity and anti-adipogenesis effects of this terpenoid.^(28,29)^

Cancer stem cells (CSCs), which have emerged as a novel target for a better understanding of chemopreventive phytochemicals, have biological and biochemical characteristics that are associated with normal stem cells, as well as an ability to induce all cell types found in cancer specimens. They are known to exist in most neoplasms and may be responsible for tumor initiation, progression, metastasis and relapse.^(30)^ One of the earliest review articles regarding the effects of phytochemicals on CSCs was presented by Kawasaki *et al.*^(31)^ in 2008, who described the possibility of these agents to modulate cancer cell growth by targeting CSCs. Dandawate *et al.*^(32)^ also found that several types of phytochemicals, including curcumin, resveratrol, tea polyphenols (EGCG, epigallocatechin), sulforaphane, genistein, indole-3-carbinol, 3,3'-di-indolylmethane, vitamin E, retinoic acid, quercetin, parthenolide, triptolide, 6-shogaol, pterostilbene, isoliquiritigenin, celastrol and koenimbin, had effects on growth of CSCs in breast tissues.

The above-mentioned novel mechanisms underlying the versatile bioactivities of phytochemicals should be targeted in future mechanistic studies. Furthermore, fundamental questions regarding why these agents exert physiological activities remain to be fully answered, since they are essentially biosynthesized by plants for self defense and not for beneficial effects in humans.

## Phytochemicals as Xenobiotics

**Fig. 2:**
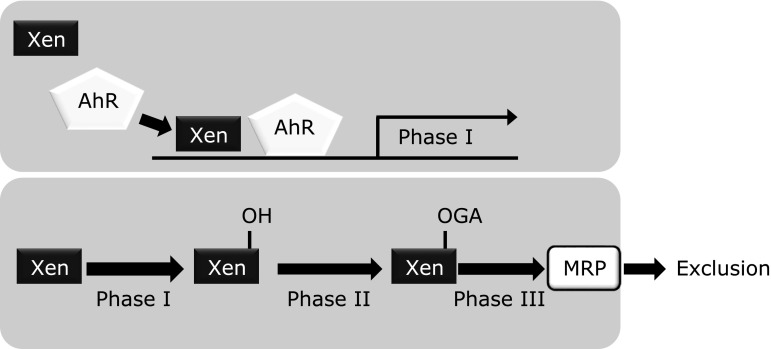
Detoxification mechanism consisting of Phase 1, 2 and 3 enzymes, and proteins. Xenobiotics are detoxified and excluded through concerted activation of enzymes and proteins in Phase 1, 2 and 3 enzymes and proteins. Most environmental xenobiotics, such as procarcinogens and dioxins, are biologically activated by Phase 1 enzymes, which give hydroxyl group(s) to them. Subsequently, chemically modified xenobiotics are provided with hydrophilic groups, including glucuronic acid. Those metabolites are then transported and excluded from the cell into the bloodstream through MRPs in an ATP-dependent manner. Generally, water solubility of the compounds increases at each stage. Xen, xenobiotics; AhR, aryl hydrocarbon receptor; GA, glucoronate; MRP, multi-drug resistant protein.

**Fig. 3:**
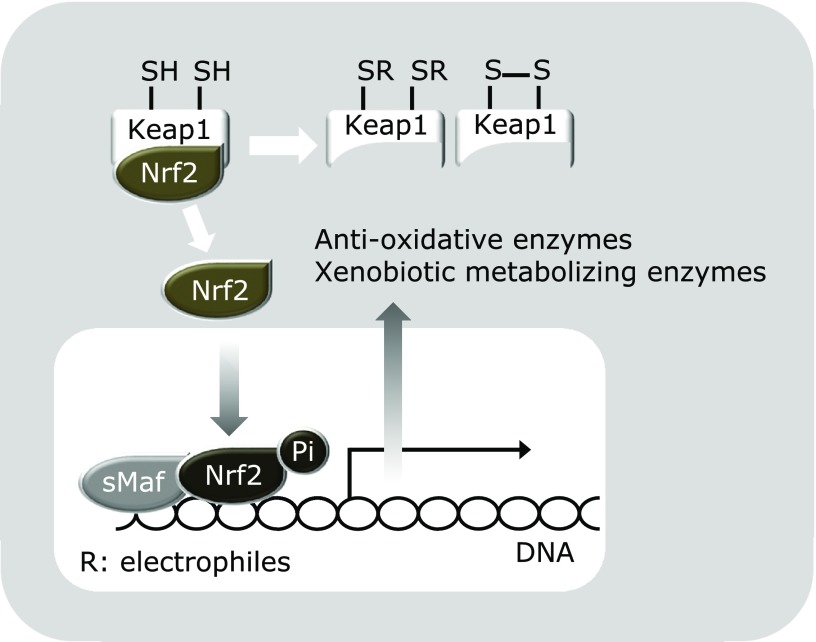
Oxidative and electrophilic stress induce Nrf2 transactivation. In the absence of environmental stress, Nrf2, a transcription factor, is inactivated because it is continuously ubiquitinated by the Cul3-Keap1 ubiquitin E3 ligase complex and thereby rapidly subjected to degradation in proteasomes. Electrophilic chemicals and oxidative stress oxidize the reactive cysteine residues of Keap1 for reducing E3 ligase activity. This critical step stabilizes Nrf2 and thereby induces robust expression of a battery of cytoprotective genes, including anti-oxidation and xenobiotics metabolizing enzymes.

As previously noted, phytochemicals are plant secondary metabolites and thus fundamentally function as xenobiotics for other organisms, including microorganisms, insects, and animals. Once these foreign chemicals gain access to tissues and cells, rapid and selective biological defense mechanisms related to xenobiotic detoxification and exclusion are activated. Xenobiotics are known to be metabolized by drug-metabolizing enzymes, which are divided into two stages; phase 1 (e.g., cytochrome P450s that adds a hydrophilic functional group) and phase 2 [e.g., glutathione (GSH)-*S*-transferase (GST), the latter of which provides GSH to that metabolized by phase 1 enzymes]. In addition, a phase 3 system promotes the conjugated metabolites to be removed from the cell by the functions of transporters such as multidrug-resistant proteins, including P-glycoprotein (Fig. 2). For example, most polycyclic aromatic hydrocarbons, termed procarcinogens, are biologically inactive in their native structure and activated by phase 1 enzymes, leading to formation of ultimate carcinogens that can bind to cellular DNA. Alternatively, activated carcinogens are subjected to reaction by phase 2 enzymes to be inactivated and then excreted from the body. Those anti-carcinogenic proteins are known to be produced via the Kelch-like ECH-associated protein 1/nuclear factor erythroid 2-related factor 2 (Keap1/Nrf2) system, which adaptively functions to protect cells from oxidative and electrophilic damages (Fig. 3).^(33)^ In a normal state, the transcription factor Nrf2 is continuously ubiquitinated by the Cul3-Keap1 ubiquitin E3 ligase complex and thereby rapidly subjected to degradation in proteasomes. Electrophilic chemicals and oxidative stress oxidize the reactive cysteine residues of Keap1 in both direct and indirect manners.^(34)^ This critical step stabilizes Nrf2, thereby inducing robust expressions of a battery of cytoprotective genes. Thereafter, P-glycoprotein, known as a plasma membrane glycoprotein, confers multidrug resistance to cells by virtue of its ability to exclude cytotoxic drugs in an ATP-dependent manner during the phase 3 detoxification stage.^(35)^

Ample evidence has been presented showing that phytochemicals are agents that markedly up-regulate those drug-metabolizing systems, indicating that these chemicals are recognized as unusable for maintaining homeostasis in animals. Their most well-known effects on drug-metabolizing systems are selective induction of the phase 2, but not phase 1, system. A pioneering work by Talalay and colleagues found that organosulfur compounds in broccoli, including sulforaphane, up-regulated the expressions of quinone reductase NAD(P)H oxidoreductase and GSTs without affecting those of P450 phase 1 enzymes.^(36)^ Importantly, such selective induction has been noted as a potential mechanism underlying chemopreventive effects demonstrated in a variety of experimental models.^(37)^ As noted above, electrophilicity is the key chemical moiety to stimulate the Keap1/Nrf2 system for inducing phase 2 enzymes, because electrophilic modification of the cysteine residues of Keap1 triggers dissociation of Nrf2 for nuclear translocation for its transcriptional activation. In support of this notion, a number of phytochemicals have this functional moiety, i.e., the *enone* structure, and are reported to activate phase 2 enzymes. For example, nordihydroguaiaretic acid, an anti-oxidative phytochemical present in the creosote bush (*Larrea tridentata*), was found to increase the level of Nrf2 protein and enhance the expression of heme oxygenase-1 (HO-1) in wild-type mouse embryo fibroblasts, but not in those from Nrf2 knockout mice.^(38)^ Furthermore, curcumin, which possesses an *enone* structure, has been demonstrated to markedly induce phase 2 enzymes in several rodent organs, including rat livers,^(39)^ and mouse livers and kidneys.^(40)^ Schneider *et al.*^(41)^ also indicated that curcumin is biochemically and chemically unstable, thus it generates several different degradation products. Intriguingly, of those degradation products, vanillin (4-hydroxy-3-methoxybenzaldehyde) and *p*-hydroxybenzaldehyde also have an electrophilic aldehyde moiety, suggesting an additional contribution to Nrf2-dependent self-protective gene expressions by curcumin. Moreover, the synthetic analog of curcumin, bis(2-hydroxybenzylidene)acetone, is more electrophilic than curcumin, and was reported to highly activate NAD(P)H quinone dehydrogenase 1 and GSH reductase in L1210 murine leukemia cells.^(42)^ Some of the terpenoids also possess this reactive moiety. Monoterpene aldehydes with fragrant properties, such as perillaldehyde and citral, were reported to up-regulate Nrf2-dependent gene expression,^(43)^ while adrographolide, a bitter diterpene lactone present in the stems and leaves of *Andrographis paniculata*, has an electrophilic moiety and was identified as an effective Nrf2 activator.^(44)^ On the other hand, some flavonoids have been shown to be converted into electrophilic *o*-quinone counterparts through auto-oxidation, suggesting that they activate Nrf2. In fact, Sriam *et al.*^(45)^ found that EGCG augmented anti-oxidative efficacy and thereby mitigated inflammatory processes during bleomycin-induced experimental pulmonary fibrosis via the Keap1/Nrf2 signaling pathway. Moreover, Lee *et al.*^(46)^ reported that piceatannol (3,4,3',5'-tetrahydroxy-trans-stilbene) derived from the seeds of *Euphorbia lagascae*, which has potential to be converted into an *o*-quinone counterpart, induced translocation of Nrf2 into nuclei and showed transcriptional activities in MCF10A human breast epithelial cells. Interestingly, they also found that the thiol reducing agents dithiothreitol and β-mercaptoethanol significantly attenuated piceatannol-induced Nrf2 activation and HO-1 expression, suggesting electrophilic addition of the putative *o*-quinone counterpart to cysteine thiol(s) of Keap1. Using matrix-assisted laser desorption ionization time-of-flight mass spectrometry, Sumi *et al.*^(47)^ demonstrated that catechol metabolites of estrogen activated Nrf2 for inducing HO-1 and the glutamate cysteine ligase catalytic subunit in RAW264.7 mouse macrophages, and also revealed that multiple reactive thiol groups of Keap1 were modified by their quinone metabolites.

In addition to electrophilic xenobiotics or phytochemicals, the Keap1/Nrf2 system is susceptible to oxidative stress, which can oxidize reactive thiol groups of Keap1 to form a disulfide bond (Fig. 3). In this context, there are numerous phytochemicals that have pro-oxidative properties. Furthermore, it should be pointed out that the above-mentioned phytochemicals, including ITCs, catechol-type flavonoids and other electrophilic compounds, are potential pro-oxidants. This notion is supported by their chemical characteristics causing them to react with intracellular anti-oxidants such as GSH, leading to its consumption. A decrease in anti-oxidant levels in cells then results in an increase in Keap1 proteins that have oxidized thiol groups for Nrf2 transactivation. Those mechanisms are quite reasonable, because Nrf2-mediated up-regulation of drug metabolizing and anti-oxidative enzymes can contribute to homeostasis by excluding xenobiotic phytochemicals and suppressing oxidative stress induced by those agents. Taken together, electrophilic phytochemicals activate Nrf2 through a dual mechanism, direct addition to Keap1 thiol groups and oxidative stress-mediated thiol oxidation of Keap1 (Fig. 3).

Up-regulation of adaptive systems by electrophilic and pro-oxidative phytochemicals may be related to the fact that they function as xenobiotics in animals. Issues related to their low bioavailability in animal models and human studies also illustrate that they are designated as ‘uninvited guests’ in humans. Most flavonoids have been reported to undergo glucuronidation, sulfation, and methylation in the small intestine and liver, while aglycone is scarcely detected in plasma.^(48)^ In contrast, conjugated metabolites are predominantly found in plasma after flavonoid ingestion and many reports have demonstrated that most, if not all, of those metabolites exhibit dramatically decreased bioactivity in comparison to their parent flavonoids.^(49,50)^ Similarly, the plasma level of ITC has been reported to be subtle after ingestion, since it is rapidly converted to a GSH conjugate by GST, and then exported from cells by multidrug resistance proteins and finally metabolized in the mercapturic acid pathway to the corresponding mercapturic acid.^(51)^ Using a rat model, Ireson *et al.*^(52)^ demonstrated that curcumin (40 mg/kg, i.v.) was not detected in plasma within 1 h of administration, while the major metabolites of curcumin were identified to be curcumin glucuronide and curcumin sulfate, with hexahydrocurcumin, hexahydrocurcuminol, and hexahydrocurcumin glucuronide also present in small amounts. Importantly, the active metabolite of curcumin in plasma has yet to be identified. These findings again raise the possibility that administered phytochemicals are rapidly converted into biologically inactive metabolites, so that the concentrations of phytochemicals themselves in plasma do not reach a level high enough to exhibit toxicity. Therefore, the low bioavailability of phytochemicals is in principle a quite reasonable phenomenon and can be recognized as a homeostatic mechanism in animals.

## Hormesis

**Fig. 4:**
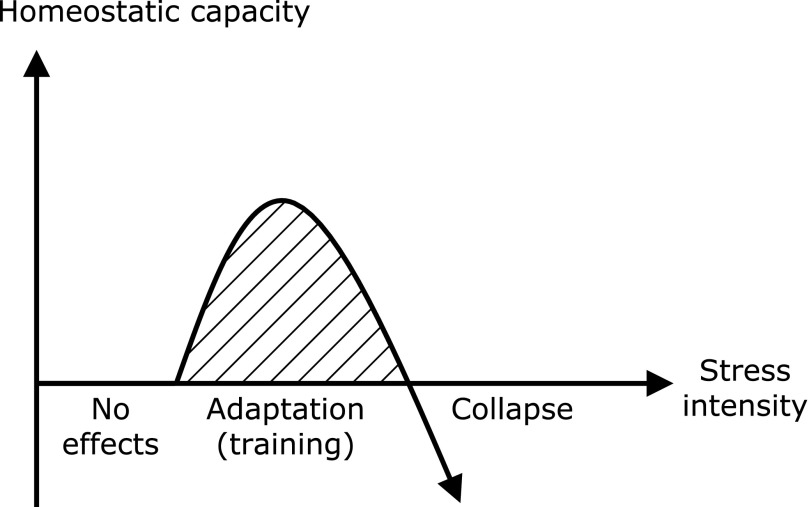
Schematic presentation of principle of hormesis. Low or moderate doses of environmental stress stimulate several adaptation systems, which, in principle, result in strengthened adaptation capacity. It is worth noting that continuous exposure to appropriate doses of stress can train adaptation machineries. However, high doses cause collapse of the adaptation machineries, as observed by death of cells, organs and organisms.

The term ‘hormesis’ was coined by C.M. Southam and J. Ehrlich in 1943 in connection with the ancient observations of H. Schulz who noticed that the growth of yeast could be stimulated by small doses of various poisons. Presently, hormesis is considered to be a unique adaptation mechanism by which cells exhibit a biphasic response to increasing amounts or levels of stress, such as from harmful chemicals, radiation, and infectious microorganisms. This unique phenomenon is often characterized by typical U- or J-shaped curves (Fig. 4). When a stressor is retained at an undetectable level, no marked changes in the adaptive mechanisms of cells or tissues occur. It is worth noting that such self-defense systems can be up-regulated and potentiated for adaptation and survival, because cytoprotective and restorative genes, such as growth and neurotrophic factors, phase 2 and antioxidant enzymes, and molecular chaperones, are notably induced. However, a catastrophic outcome is readily predictable when stress strength exceeds the defense capacity of the cell, tissue, or organism. In 1987, Calabrese *et al.*^(53)^ published an overview regarding occurrence of hormesis by different chemical classes (heavy metals, polycyclic aromatic hydrocarbons, others), based on the affected biological and toxic endpoints, such as growth enzyme activities, DNA repair capacity, lifespan and tumor incidence, and by biological/taxonomic systems. Also, dietary restriction, a factor contributing to lifespan extension, is associated with hormesis, with sustained moderate hyperadrenocorticism underlying that effect.^(54)^ There is also accumulating evidence that many phytochemicals exhibit hormesis-related phenomena, with modulation of the Keap1/Nrf2 system a representative example. Pall and Levine^(55)^ proposed a hypothesis stating that the most healthful diets known, traditional Mediterranean and Okinawan, are rich in Nrf2-activting nutrients, as was the Paleolithic diet in ancient times, while modern diets are poor in such nutrients. ITCs, well-known Nrf2 activators,^(56)^ have exhibited toxicity in rodents when given at high doses.^(57)^ As noted above, catechol-type polyphenols are potential pro-oxidants based on their chemical property that allows them to undergo auto-oxidation. Also, Lambert *et al.*^(58)^ demonstrated that a high oral dose of EGCG exhibited hepatotoxicity in mice, which is consistent with this notion. Along with that observation, we previously found that a commercial mixture of green tea polyphenols significantly aggravated dextran sulfate sodium (DSS)-induced acute colitis and tended to promote adenocarcinoma formation in mice.^(59)^ On the other hand, low and medium (0.01–0.1% in diet), but not high (1%) doses of green tea polyphenols ameliorated DSS-induced hepatotoxicity and nephrotoxicity.^(60)^ Collectively, the effects of EGCG and green tea polyphenols on several different organs in rodents are dependent on dose and occur in a hormetic manner.^(61)^ Demirovic and Rattan reported that curcumin modulated wound healing in a biphasic dose response manner *in vitro*, as it was stimulatory at low (1–5 µM) and inhibitory at higher concentrations.^(62)^ In addition, curcumin at very low concentrations (~1 µM) shows anti-oxidative effects, while it functions primarily as an autophagy inducer at medium concentrations (5–10 µM).^(63)^ Similarly, curcumin treatment (1 µM) increased proteasome activity (chymotrypsin-like activity) by 46% as compared to that in untreated keratinocytes, while higher concentrations (>1 µM) were inhibitory.^(64)^ Interestingly, Proshkina *et al.*^(65)^ found that short-term treatment with flavonoids [quercetin and (–)-epicatechin] had beneficial effects, and conferred resistance to paraquat and acute γ-irradiation in *Drosophila melanogaster*, whereas long-term treatment did not change or even decreased lifespan.

## Non-specific Protein Binding by Zerumbone

**Fig. 5:**
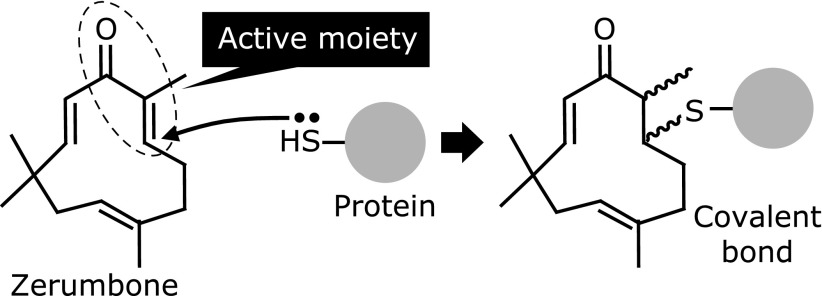
Chemical structure of zerumbone and its electrophilic addition to protein thiol moieties to form a covalent bond.

In results obtained with a variety of rodent models, we have shown that zerumbone, a sesquiterpene present in *Zingiber zerumbet* Smith, is an effective anti-oxidative, anti-inflammatory and chemopreventive agent.^(66,67)^ It possesses two different modes of actions to exhibit those activities. First, zerumbone is able to suppress the expression of cyclooxygenase-2 (COX-2), a rate-limiting enzymes present in most pro-inflammatory and oncogenic processes.^(68,69)^ An intriguing finding is that zerumbone did not have an effect on lipopolysaccharide (LPS)-triggered activation of mitogen-activated protein kinases (MAPKs; extracellular signal-regulated kinase 1/2, c-Jun N-terminal kinase1/2, p38MAPK) or key transcription factors (activator protein-1 and nuclear factor kappaB; NF-κB), while it targeted a post-transcriptional mechanism.^(70)^ The stability of COX-2 mRNA has been shown to be tightly regulated by numerous proteins that are associated or dissociated with AU-rich elements in the 3'-UTR.^(71)^ Also, a COX-2 mRNA AU-rich element-binding protein, Hu-antigen R (HuR), has been shown to have a detrimental effect on COX-2 mRNA half-life.^(72)^ In another study, we prepared sepharose beads, which zerumbone bound to.^(73)^ A competitive pull-down assay of cell lysate from RAW264.7 mouse macrophages incubated with zerumbone-bound sepharose beads with or without increased concentration of zerumbone was then conducted. Western blot analysis of the HuR protein in the pull-down fraction suggested that zerumbone binds to this ARE-binding protein. However, a later experiment using a biotin-derivative of zerumbone disclosed that HuR is not a binding protein of zerumbone in living RAW264.7 cells (Ohnishi *et al.*, unpublished observation). Another essential mechanism identified as underlying the anti-oxidative, anti-inflammatory, and chemopreventive activities of zerumbone is activation of the Nrf2 system. This agent is capable of increasing the expressions of several Nrf2-dependent genes in cellular and rodent models.^(74–79)^ It is also important to note that zerumbone increased the expression of HO-1 in wild-type, but not Nrf2 knockout mice.^(75)^ Its property of binding to Keap1 is predictable since it has an electrophilic, α,β-unsaturated carbonyl group, as mentioned above (Fig. 5). Consistent with this notion, it was found that a biotin-derivative of zerumbone exhibited the highest binding affinity to Keap1 among 8 key proteins (MAPKs, transcription factors, others) in LPS-stimulated signal transduction pathways (Ohnishi *et al.*, unpublished observation). Therefore, Keap1 has been identified as a critical molecular target of zerumbone to exhibit bioactivity.

It is tempting to speculate that zerumbone has other binding proteins on account of its low molecular weight (MW: 218) and simple chemical structure. That may be supported by the observations of Eaton *et al.*^(80)^ that 4-hydroxy-2-nonenal, a degradation product of lipid peroxides with a low molecular weight (MW: 156) and electrophilicity, bound to a number of proteins in rat hearts and rat aortic smooth-muscle cells.^(81)^ To explore the broad protein binding range, we generated a novel antibody able to specifically recognize zerumbone adduct proteins.^(82)^ Treatment of Hepa1c1c7 mouse hepatoma cells with that agent resulted in a dramatic increase of those proteins in a time-dependent manner. Thereafter, immunocytochemistry experiments that used this antibody revealed that those adduct proteins were broadly localized in the cytoplasm and nucleus within 30 min after starting incubation. Such extensive protein modification by small molecules disrupts the stereo-structure and functions of cellular proteins. In fact, zerumbone was found to increase E3 ubiquitin-protein ligase-dependent protein ubiquitination, a hallmark of protein denaturation and formation of aggesome,^(82)^ a cytoplasmic structure containing misfolded proteins. Such a proteo-stress nature is considered to be a side-effect of zerumbone with beneficial physiological functions. Interestingly, cells in which zerumbone adducts were loaded were found to activate protein quality control systems for homeostasis, which is comprised of molecular chaperones (induction of heat shock proteins, HSPs), proteasome (increases in b5 expression and chymotryptic activity), and autophagy (increases in expressions of pro-autophagic genes, including p62).^(82,83)^ A concerted increase by defense systems against proteo-toxic stimuli led to phenotypic change in zerumbone-exposed cells. For example, zerumbone addition upregulated small HSP mRNAs and conferred a thermo-resistant phenotype to the nematodes *Caenorhabditis elegans.*^(82)^ It is also worth noting that mild heat pretreatment of the nematodes was protective against heat shock. Moreover, Hepa1c1c7 cells pretreated with zerumbone were found to be more resistant to 4-hydroxy-2-nonenal-induced protein modifications and cytotoxicity. Collectively, non-specific protein modifications by zerumbone can be perceived as phenomena related to hormesis and not merely side-effects, as long as the level of proteo-stress remains within a tolerable range.

## Unique Role of Proteo-stress in Anti-inflammatory Functions

**Fig. 6:**
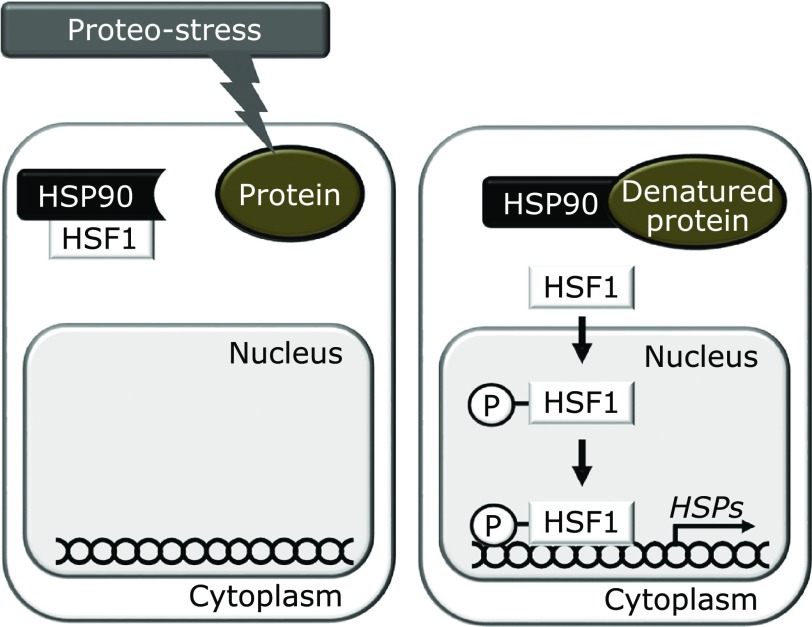
Mechanisms of HSP induction via key transcription factor HSF1. Proteo-stress, such as heat shock and electrophiles, increases denatured proteins, which are bound to HSP90, the major molecular chaperone in the cytoplasm. This leads to dissociation of HSF1 from HSP90 and phosphorylated HSF1 translocating into the nucleus for transactivation, after which it targets a variety of genes involved in protein quality control, e.g., HSPs.

Subsequently, we attempted to find an association of the proteo-stress property of zerumbone with its mechanisms of anti-inflammatory actions. This seemed to be relevant, because heat shock responses, including induction of HSPs, have been reported to be associated with anti-inflammatory mechanisms.^(84)^ For example, heat shock factor 1 (HSF1), activated by proteo-stress stimuli such as heat shock, is the master transcription factor responsible for numerous chaperone molecules, including HSPs (Fig. 6). Both Nrf2 and HSF1 are currently recognized as major transcription factors for cytoprotection,^(85)^ and there is ample evidence that HSF1 acts as an anti-inflammatory transcription factor with several different mechanisms. Wu *et al.*^(86)^ found that HSF1 attenuated tumor necrosis factor (TNF)-α-induced cardiomyocyte death through suppression of the NF-κB pathway, in which HSF1-induced HSP70/HSP90 disrupted the translocation of the NF-κB component RelA, while HSP70 was reported to inhibit the LPS-induced NF-κB pathway by interacting with TNF receptor-associated factor 6, a crucial signaling transducer for NF-κB activation.^(87)^ In addition, the proximal TNF-α promoter/5'-UTR sequence was found to compete for HSF1 binding to a classic heat shock element and overexpression of HSF-1 decreased promoter activity of the TNF-α gene.^(88)^ On the other hand, a recent report by Zhang *et al.*^(89)^ showed that HSF1 over-expression augmented induction of an anti-inflammatory cytokine, interleukin IL-10 mRNA.

The findings noted above led us to examine the effects of zerumbone on HSF1 and its contribution to its bioactivities to clarify the role of proteo-stress. As expected, treatment of RAW264.7 macrophage with zerumbone led to marked nuclear translocation of HSF1 for HSP70 protein expression.^(90)^ Interestingly, pretreatment with *N*-acetyl-l-cysteine (competitive nucleophile that reacts with zerumbone) or 4-phenylbutyric acid (chemical chaperone^(91)^) reduced the suppressive effects of zerumbone on LPS-induced inducible nitric oxide synthase and COX-2 expression. Similarly, the suppressive effects of zerumbone on LPS-induced pro-inflammatory gene expressions were significantly diminished in HSF1-downregulated cells. Those results strongly suggest that the non-specific protein binding of zerumbone has a unique and significant role in anti-inflammatory activities through activation of HSF1.

## Conclusions

**Fig. 7:**
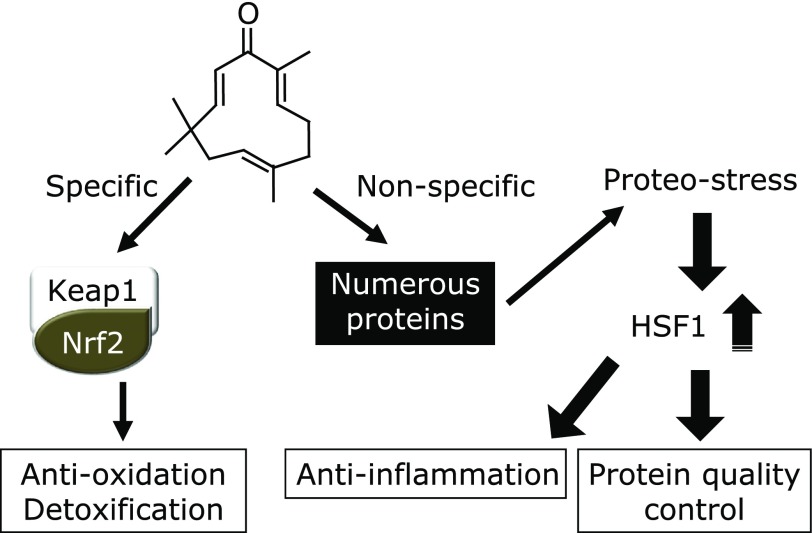
Dual mechanisms underlying biological functions of zerumbone. Zerumbone specifically targets Keap1 to induce Nrf2 activation, leading to transcriptional activation of self-protective genes, such as anti-oxidation and detoxification. Alternatively, this agent binds with many cellular proteins in a non-specific manner, which may result in increased proteo-stress. If proteo-stress by zerumbone remains moderate, HSF1 is activated to induce a variety of genes involved in protein quality control, e.g., HSPs. Importantly, zerumbone partially exhibits its anti-inflammatory functions through HSF1. Thus, both specific and non-specific interactions of zerumbone significantly contribute to its biological functions.

In this review article, novel and unique dual mechanisms underlying the biological and physiological activities of zerumbone are highlighted (Fig. 7). A quite essential question is whether other phytochemicals exert their functions via similar mechanisms. Although further investigations are needed, we have found that many of the phytochemicals tested were able to up-regulate HSP70 in mouse hepatoma cells, while nutrients were mostly inactive.^(82)^ Unlike synthetic drugs, phytochemicals are designed and biosynthesized in a way to function in plants for adaption to environmental stress, thus there seems to be no necessity of exerting beneficial functions in animals. Nevertheless, a quite puzzling and intriguing fact is that animal cells occasionally express specific target proteins in response to certain phytochemicals. Some may argue that the presence of such receptors is inevitable, as animal cells have evolved to express phytochemical receptors or transporters to more efficiently utilize those functional plant metabolites. Alternatively, it is speculated that some phytochemicals accidentally interact with animal proteins, which exhibit structural diversity. In any case, accumulating evidence showing the functionality and potential toxicity of phytochemicals verifies the notion that they are fundamentally xenobiotic in animals, thus appropriate dosing should be taken into account in order to achieve optimum efficacy and minimum toxicity. Another perspective would be that chronic ingestion of phytochemicals may be described as ‘chemical training’, whereby defense systems manage to induce or amplify protective mechanisms in response to xenobiotic stresses. This novel concept has mechanistic similarities with physical training and mental training since the doses of phytochemicals are critical determinants to achieve beneficial functions and potential side-effects while moderate strength of physical and mental stressors could amplify the protective systems. In addition, it is needless to say that overdoses must result in collapse of those systems. Thus it is tempting to assume that chronic exposures to phytochemicals in vegetables and fruits may be described as chemical training that can potentiate self-defense systems against harmful chemicals and endogenous stress stimuli as well. If a hypothesis based on that speculation could be proven, supplementation of phytochemicals at high doses may be life-threatening for individuals who have not been chemically trained by adequate consumption of vegetables and fruits.

## Abbreviations

AR: androgen receptor
COX-2: cyclooxygenase-2
CSC: cancer stem cells
DNMT: DNA methyltransferase
DSS: dextran sulfate sodium
EGCG: (–)-epigallocatechin-3-gallate
GSH: glutathione
GST: glutathione-*S*-transferase
HAT: histone acetyltransferases
HO-1: heme oxygenase-1
HSF1: heat shock factor 1
HSP: heat shock protein
HuR: Hu-antigen R
*hTERT*: human telomerase reverse transcriptase
ITC: isothiocyanate
Keap1: Kelch-like ECH-associated protein 1
LPS: lipopolysaccharide
MAPK: mitogen-activated protein kinase
MRP: multi-drug resistant protein
NF-κB: nuclear factor kappaB
Nrf2: nuclear factor erythroid 2-related factor 2
SOCS: suppressors of cytokine signaling
TNF: tumor necrosis factor
UTR: untranslated region
UV: ultraviolet
